# Integrative Metabolomic and Echocardiographic Profiling Reveals Metabolic–Cardiac Structural Coupling in Yili Horses During Incremental Exercise

**DOI:** 10.3390/ani16111672

**Published:** 2026-05-30

**Authors:** Xiaokang Chang, Jiangfei Peng, Zihan Zhang, Manjun Zhai, Hongzhong Chu, Runchen Yao, Penghui Luo, Xinkui Yao, Wanlu Ren, Yaqi Zeng

**Affiliations:** 1 College of Animal Science, Xinjiang Agricultural University, Urumqi 830052, China; changxk312@126.com (X.C.); pengjiangfei1013@126.com (J.P.); zhangzihan0809@126.com (Z.Z.); zhaimanjun@yeah.net (M.Z.); yxk61@126.com (X.Y.); renwanlu@xjau.edu.cn (W.R.); 2 Xinjiang Key Laboratory of Equine Breeding and Exercise Physiology, Urumqi 830052, China; 3 Xinjiang Yili Kazakh Autonomous Prefecture Animal Husbandry Station, Yining 835099, China; 13364712998@163.com (H.C.); m18095936088@163.com (R.Y.); 4 Animal Husbandry Station of Xinjiang Uygur Autonomous Regio, Urumqi 830004, China; xmzztxzx1@163.com

**Keywords:** Yili horse, incremental exercise, metabolomics, cardiac structure, coupling

## Abstract

This study integrated echocardiography with widely targeted metabolomics to decipher how plasma metabolic dynamics couple with cardiac geometry in Yili horses during incremental treadmill exercise. Nine speed-type horses underwent a graded exercise test (6% incline; 0 to 9 m/s). Jugular venous blood samples collected at rest (0 m/s) and at 3, 5, 7, and 9 m/s were profiled by LC-MS, and Pearson correlation analysis was applied to relate differentially expressed metabolites (DEMs) to twenty echocardiographic structural indices. A core set of 314 shared DEMs (124 upregulated, 190 downregulated) was identified across all exercise comparisons, spanning amino acids, organic acids, and fatty acyls. These metabolites were mapped to ABC transporter, thermogenesis, aldosterone-regulated sodium reabsorption, steroid hormone biosynthesis, and one-carbon folate metabolism pathways. At rest (0 m/s), right ventricular end-diastolic dimension correlated positively with arginyl-isoleucine (*p* < 0.001), whereas left ventricular free wall thickness (diastolic and systolic) correlated positively with undecanedioic acid (*p* < 0.001) and proline-hydroxyproline (*p* < 0.01). At peak exercise (9 m/s), left ventricular mass and left ventricular mass index correlated positively with succinic acid (*p* < 0.05) and methylmalonic acid (*p* < 0.05), while left ventricular minor axis correlated with carnitine C14:2 and carnitine C12:1 (*p* < 0.05). Left ventricular end-systolic dimension and left atrial end-diastolic dimension correlated negatively with cysteine-glutathione disulfide and N2-(1-carboxyethyl)-L-arginine, respectively. These findings illuminate a robust metabolic–cardiac structure axis: amino acid metabolites support collagen matrix turnover and redox homeostasis, organic acids sustain mitochondrial energy flux and antioxidant defense, and fatty acyls fuel continuous contractile activity via enhanced fatty acid oxidation. This metabolome-informed framework furnishes a mechanistic basis for precision training and performance phenotyping in equine athletes.

## Simple Summary

This study explores how blood chemical changes relate to heart shape in Yili horses during progressively harder exercise. Horses ran on a treadmill at increasing speeds while researchers used ultrasound to examine their hearts and analyze small molecules in their blood. The results showed that hundreds of blood chemicals changed significantly during exercise, and many of these were closely linked to heart wall thickness and chamber size, playing roles in energy supply and tissue protection. These findings suggest that blood testing can reflect heart function in horses, providing new guidance for training Yili horses and evaluating their athletic ability.

## 1. Introduction

Incremental treadmill exercise testing, which systematically monitors physiological indices such as lactate, blood gases, and heart rate through graded speed increments, has been widely adopted for determining anaerobic threshold and evaluating exercise tolerance in equine athletes [1]. Since its introduction in the late twentieth century, the equine treadmill has emerged as a cornerstone of equine exercise physiology research, offering a controlled and highly reproducible environment for precisely characterising physiological responses across varying workloads [2]. Its application has since expanded well beyond rehabilitative protocols to encompass fitness conditioning, gait analysis, cardiac functional assessment, and athletic performance profiling [3]. Substantial evidence indicates that increasing treadmill incline enhances oxygen delivery to working muscles by augmenting venous return and cardiac output [4], while distinct running speeds elicit markedly divergent metabolic responses during high-intensity efforts in horses [5].

The heart represents the principal determinant of athletic endurance, rendering its structural and functional evaluation critical for assessing equine athletic capacity. Echocardiography stands as the most extensively applied non-invasive technique for quantifying cardiac dimensions and performance in horses [6], although its measurements are substantially influenced by breed, age, sex, body size, and training history [7,8]. Systematic training induces physiological cardiac remodelling in horses, characterised by ventricular wall thickening, chamber enlargement, and increased cardiac output [9]. Nevertheless, existing investigations have been largely restricted to descriptive accounts of cardiac structure under resting conditions, creating a substantial knowledge gap regarding the interplay between cardiac geometry and metabolic dynamics during incremental exercise.

Blood indices provide a window into the physiological fluctuations that accompany training and competitive exertion. Erythrocyte count, haemoglobin concentration, and packed cell volume govern oxygen transport capacity, while lactate accumulation, pH decline, and blood gas perturbations signal the shift from aerobic to anaerobic metabolism [10]. Indeed, peak haematocrit, nadir pH, and lactate threshold velocity obtained during incremental exercise tests correlate strongly with racing performance [11]. However, these conventional indices merely snapshot acute systemic stress; they fall critically short of exposing the molecular mechanisms that drive exercise adaptation.

Metabolomics is a systems-level approach to studying small-molecule metabolites that reveals how organisms integrate genetic background and environmental cues into a unified physiological response [12]. Within exercise science, metabolomics has emerged as a pivotal bridge linking external training stimuli to metabolic pathways and resultant physiological phenotypes [13]. Exercise training markedly alters the blood metabolite repertoire in an intensity- and duration-dependent manner [14]. In equines, this translational potential is becoming increasingly apparent: both training status and exercise intensity trigger substantial shifts in plasma and skeletal muscle metabolic profiles [15], systematic conditioning enhances maximal aerobic capacity alongside sustained skeletal muscle metabolomic remodelling [16], and prolonged endurance competition profoundly perturbs circulating lipid and amino acid signatures [17]. Nevertheless, these investigations have largely confined their lens to isolated time points or discrete exercise intensities, leaving the continuous metabolic trajectory across a full incremental exercise protocol largely uncharted.

The Yili horse is a Chinese breed developed through selective breeding, renowned for its superior endurance capacity and speed adaptability, and occupies an important position in domestic equestrian sports and the racing industry [18,19]. This breed has long been cultivated in the high-altitude, hypoxic environment of the Ili grassland, developing distinctive cardiopulmonary and energy metabolic phenotypes. Nevertheless, despite its widely recognized athletic potential, systematic exercise physiology research on this breed remains scarce, particularly studies integrating echocardiography with metabolomics as a multidimensional evaluation approach.

From an integrative physiology perspective, cardiac geometric parameters govern systemic pumping capacity and the upper limit of aerobic endurance [20,21], whereas plasma metabolite profiles under incremental workloads reflect the molecular status of energy substrate utilization, oxidative stress defence, and adaptive regulation in cardiac and skeletal muscle [22,23]. There likely exists an intrinsic “structure-function coupling” between the static cardiac structural phenotype captured by echocardiography and the dynamic metabolic response revealed by metabolomics. This hypothesis, however, has not been validated in equine models, and quantitative evidence specific to the Yili horse is particularly lacking.

To address these research gaps, the present study employed a treadmill incremental exercise model combined with echocardiography and broad-targeted metabolomics in nine speed-type Yili horses, aiming to elucidate the dynamic characteristics of blood metabolomics during incremental exercise and their association mechanisms with cardiac structure, thereby providing a theoretical basis for scientific training and athletic performance evaluation in this breed.

## 2. Materials and Methods

### 2.1. Animals

This study was approved by the Animal Welfare and Ethics Committee of Xinjiang Agricultural University (approval number: 2023037). Nine speed-type Yili horses (*n* = 9), bred at the Zhaosu Horse Farm in the Yili Kazakh Autonomous Prefecture, Xinjiang, China, were enrolled in this study. The sample size was determined based on convenience sampling. All horses were 2 years old with a body weight of 360.9 ± 24.8 kg (detailed baseline information is provided in Table S1). Prior to enrollment, each horse underwent a comprehensive clinical veterinary examination to confirm good health status, including a resting heart rate < 45 beats/min, rectal temperature < 38.5 °C, and absence of lameness or respiratory disease. Before the commencement of the study, all horses received a standardized 3-month conditioning training program to ensure their capability to complete the treadmill incremental exercise protocol. Horses meeting any of the following criteria were excluded: (1) occurrence of exercise-induced injury or illness during the conditioning period; (2) failure to complete the entire incremental load protocol during treadmill testing; (3) detection of abnormalities on resting echocardiography; (4) incomplete blood sample collection at any time point; or (5) administration of any medication within 2 weeks prior to the study. Husbandry and management practices were strictly performed in accordance with the protocols described by Li et al. [24].

### 2.2. Experimental Procedures and Sample Collection

#### 2.2.1. Measurement and Analysis of Cardiac Structural Parameters

Resting echocardiographic examinations were performed using a Mindray M6 veterinary portable colour Doppler ultrasound system equipped with a 2.5 MHz phased-array transducer (Mindray, Shenzhen, China). The transducer was placed on the right hemithorax between the third and fourth intercostal spaces. Two-dimensional (2D) and M-mode images were acquired at an imaging depth of 30 cm, focal depth of 5 cm, and sector angle of 110°. All scans were conducted by a single experienced operator, and images were recorded only when the heart rate was between 32 and 45 beats/min. Each parameter was quantified over three consecutive cardiac cycles and averaged. A total of 20 cardiac structural and functional parameters were obtained (Table 1). Data were compiled using Microsoft Excel 2021 and are expressed as mean ± standard deviation (SD).

#### 2.2.2. Blood Sample Collection

**Figure 1:**
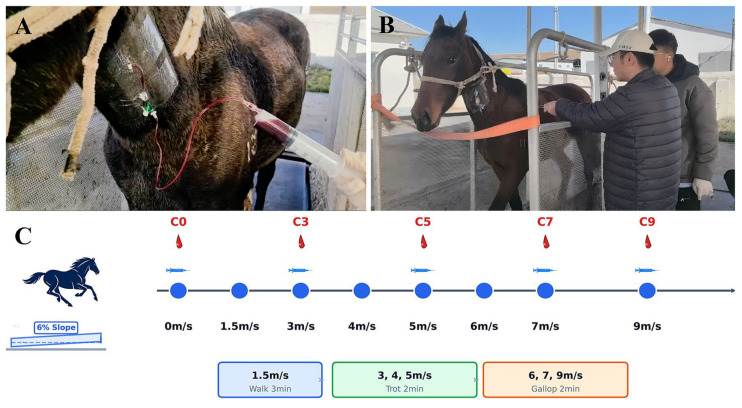
Schematic diagram of blood collection for incremental treadmill exercise. Note: (**A**) blood collection before incremental exercise; (**B**) blood collection during incremental exercise; (**C**) schematic diagram of blood sample collection protocol (used Kimi K2.6 to create).

Jugular venous blood was drawn at rest prior to exercise to serve as the baseline control (0 m/s; C0; Figure 1A). The incremental treadmill exercise protocol was conducted at a fixed 6% incline with staged speed increments: walking at 1.5 m/s for 3 min, followed by trotting at 3, 4, and 5 m/s for 2 min each, and then cantering at 6, 7, and 9 m/s for 2 min each. Within the final 30 s of the 3 m/s (C3), 5 m/s (C5), 7 m/s (C7), and 9 m/s (C9) stages, 10–15 mL of blood was collected through an indwelling 18 G intravenous catheter (Introcan^®^ Classic; catalog no. 4252098B; B. Braun Melsungen AG, Melsungen, Germany) coupled to a 30 mL sterile syringe (catalog no. 60019178; KDL Medical, Shanghai, China) (Figure 1B,C). The syringe was replaced immediately after each draw. Samples were transferred into 5 mL EDTA-K2 anticoagulant tubes and centrifuged at 3000 rpm for 10 min at 4 °C. Plasma was promptly isolated, snap-frozen in liquid nitrogen, and stored at −80 °C until widely targeted metabolomic analysis.

#### 2.2.3. Metabolite Extraction

Plasma samples were thawed on ice and vortexed for 10 s. A 50 μL aliquot of each sample was transferred to a 2 mL centrifuge tube and mixed with 300 μL of ice-cold extraction solution (acetonitrile: methanol, 1:4, *v*/*v*) containing internal standards. The mixture was vortexed for 3 min and centrifuged at 12,000 rpm for 10 min at 4 °C. The supernatant (200 μL) was collected and incubated at −20 °C for 30 min to allow further precipitation, followed by a second centrifugation at 12,000 rpm for 3 min at 4 °C. The clarified supernatant (180 μL) was retained for LC-MS analysis. For quality control (QC), equivalent volumes of supernatant from all extracted samples were pooled. Data acquisition was performed using an ExionLC AD ultra-high-performance liquid chromatography system coupled to a TripleTOF 6600 high-resolution mass spectrometer (AB SCIEX, Framingham, MA, USA) [25]. Details of the reagents used in the experiment are provided in Table S2.

#### 2.2.4. Chromatographic Separation Conditions

Samples were analyzed using three LC-MS methods. For Method 1 (T3 positive-ion mode), chromatographic separation was performed on a Waters ACQUITY UPLC HSS T3 C18 column (1.8 µm, 2.1 mm × 100 mm). Mobile phase A consisted of 0.1% (*v*/*v*) formic acid in water, and mobile phase B consisted of 0.1% (*v*/*v*) formic acid in acetonitrile. Gradient elution was executed as follows: 5–20% B (0–2 min), 20–60% B (2–5 min), 60–99% B (5–6 min), held at 99% B for 1.5 min, returned to 5% B over 0.1 min (6.0–6.1 min), and equilibrated at 5% B for 2.4 min. The column temperature was maintained at 40 °C, the flow rate was 0.4 mL/min, and the injection volume was 2 µL. For Method 2 (T3 negative-ion mode), identical chromatographic conditions were employed with detection performed in negative-ion mode. For Method 3 (HILIC negative-ion mode), separation was achieved on a Waters ACQUITY UPLC BEH HILIC column (1.7 µm, 1 mm × 150 mm). Mobile phase A was 80% acetonitrile, 10% water, and 10% methanol containing 20 mM ammonium formate (pH 10.6), and mobile phase B was 40% acetonitrile and 60% water containing 20 mM ammonium formate. The gradient elution program was: 5–20% B (0–2 min), 20–70% B (2–3.5 min), 70–95% B (3.5–6.5 min), held at 95% B for 1 min.

#### 2.2.5. Mass Spectrometry Acquisition Parameters

Data were acquired in information-dependent acquisition mode under the control of Analyst TF 1.7.1 software (SCIEX, Concord, ON, Canada). Ion source settings were as follows: GAS1 and GAS2 at 50 psi each; curtain gas at 25 psi; and source temperature at 550 °C. Declustering potential was set to +60 V in positive-ion mode and −60 V in negative-ion mode, while the ion spray voltage floating was +5000 V (positive) and −4000 V (negative).

For the TOF MS survey scan, the mass range was set to *m*/*z* 50–1000 with an accumulation time of 200 ms, and dynamic background subtraction was enabled. Product ion scans were recorded over *m*/*z* 25–1000 with an accumulation time of 40 ms. Collision energy was set to +30 V (positive)/−30 V (negative) with a collision energy spread of 15. The resolution was set to UNIT, the charge state to 1, the intensity threshold to 100 cps, and the mass tolerance to 50 ppm. Isotope ions within 4 Da were excluded, and the maximum number of candidate ions monitored per cycle was 18.

#### 2.2.6. Metabolomic Data Processing and Statistical Analysis

Raw LC-MS data were processed using MS-DIAL v4.9 for peak detection, retention time alignment, deisotoping, and normalization. Metabolite identification was achieved by matching accurate mass, isotopic distribution, and tandem mass spectral fragmentation patterns against the METLIN, Human Metabolome Database, and Kyoto Encyclopedia of Genes and Genomes (KEGG) databases. Prior to multivariate analysis, the data matrix was subjected to total ion current normalization followed by unit variance scaling. Unsupervised principal component analysis (PCA) was performed using the prcomp function in R v4.1.2 [26]. Supervised orthogonal partial least squares discriminant analysis (OPLS-DA) was conducted using the MetaboAnalystR package v1.0.1 [27]; variable importance in projection (VIP) scores were extracted to evaluate metabolite importance.

Statistical significance was assessed using Student’s t-test, and the resulting *p* values together with fold change (FC) values were used to evaluate the significance and magnitude of differential abundance. DEMs were selected according to the following thresholds: VIP > 1, *p* < 0.05, and FC ≥ 1.5 or FC ≤ 0.667. Given the limited sample size of this study, Cliff’s delta (δ) was further employed as a non-parametric effect size measure to comprehensively evaluate the practical significance of differential metabolites and quantify the magnitude of between-group differences. Cliff’s delta is calculated based on the pairwise stochastic dominance between two groups—the probability that a randomly selected observation from one group exceeds a randomly selected observation from the other. Its values range from −1 to +1, with the absolute value indicating effect magnitude: 0.147 ≤ |δ| < 0.330 denotes a small effect, 0.330 ≤ |δ| < 0.474 a medium effect, and |δ| ≥ 0.474 a large effect. To ensure reliable estimation of precision under small-sample conditions, 95% confidence intervals (CIs) for Cliff’s delta were derived via bootstrap resampling (5000 iterations). Finally, pathway enrichment analysis of the DEMs was performed using the KEGG database (http://www.kegg.jp/kegg/pathway.html, accessed on 26 March 2025).

#### 2.2.7. Correlation Analysis Between Cardiac Structure and Differential Metabolites

To evaluate the associations between cardiac structural parameters and DEMs under incremental exercise loads, Pearson correlation coefficients (r) were computed using the WGCNA package v1.69 in R v3.5.1. Significant associations were defined by the thresholds *p* < 0.05 and |r| > 0.6. Based on these significant correlations, a correlation heatmap was generated for visualization using the corrplot package v0.92 in R v4.1.2 [28].

## 3. Results

### 3.1. Cardiac Structural Parameters

Echocardiographic measurements were completed in all nine Yili horses. The raw data were compiled to yield descriptive statistics, including mean values, SD and coefficients of variation (CV), for each cardiac structural index. A summary of the cardiac structural characteristics is provided in Table 2.

### 3.2. Metabolomic Data Quality Control

**Figure 2:**
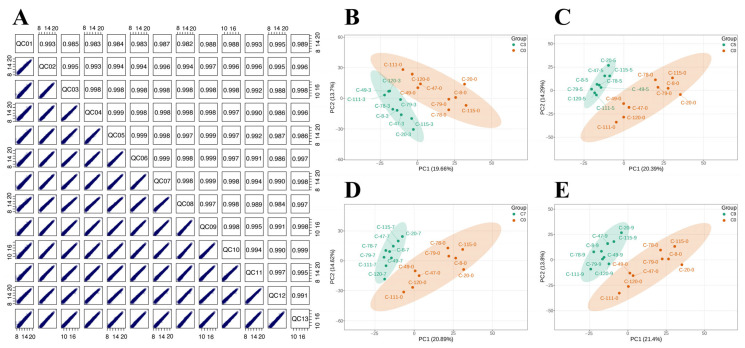
QC sample correlation and PCA score plots. Note: (**A**) QC sample correlation plot; (**B**) PCA score plot of C3 vs. C0; (**C**) PCA score plot of C5 vs. C0; (**D**) PCA score plot of C7 vs. C0; (**E**) PCA score plot of C9 vs. C0.

To ensure data reliability, thirteen QC samples were evenly interspersed throughout the analytical sequence. As shown in Figure 2A, Pearson correlation coefficients (R^2^) computed from the QC samples all exceeded 0.98, satisfying the accepted quality criterion for metabolomic analyses (R^2^ > 0.95) and confirming both instrument stability and data reproducibility. On the basis of these results, the 45 plasma samples were subjected to subsequent multivariate statistical analyses, including PCA and OPLS-DA.

### 3.3. Principal Component Analysis

Unsupervised PCA of the score plots for pairwise comparisons (C3, C5, C7, and C9 versus C0) revealed progressively pronounced separation between exercise and resting groups along PC1. The discrimination intensified with increasing workload, shifting from partial overlap at the lowest speed (C3 vs. C0; PC1 variance = 19.66%) to complete separation at the highest speed (C9 vs. C0; PC1 variance = 21.4%), with non-overlapping 95% confidence intervals (Figure 2B–E).

### 3.4. Supervised Classification by OPLS-DA

**Figure 3:**
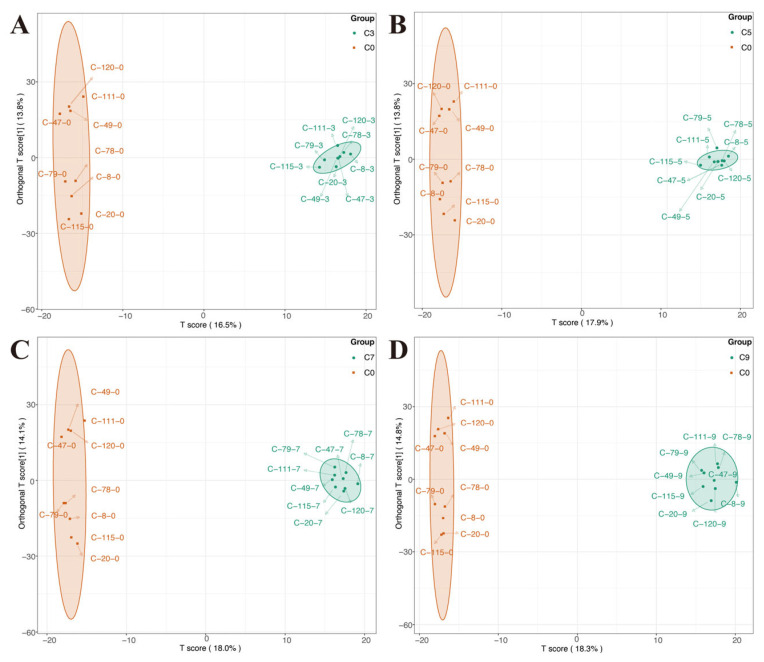
OPLS-DA score plot. Note: (**A**) OPLS-DA score plot of C3 vs. C0; (**B**) OPLS-DA score plot of C5 vs. C0; (**C**) OPLS-DA score plot of C7 vs. C0; (**D**) OPLS-DA score plot of C9 vs. C0.

To refine the group separation evident from PCA, supervised OPLS-DA was performed. Score plots for the pairwise comparisons of C3, C5, C7, and C9 versus C0 (Figure 3A–D) exhibited clear discrimination along the predictive component axis, with non-overlapping 95% confidence regions between groups and tight within-group clustering, indicative of robust and stable model discriminatory capacity.

**Figure 4:**
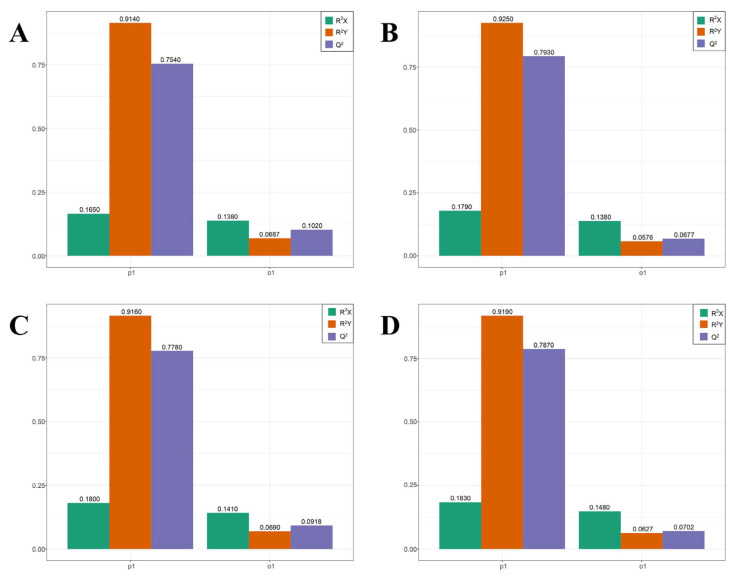
OPLS-DA model validation plots. Note: (**A**) OPLS-DA model validation plot of C3 vs. C0; (**B**) OPLS-DA model validation plot of C5 vs. C0; (**C**) OPLS-DA model validation plot of C7 vs. C0; (**D**) OPLS-DA model validation plot of C9 vs. C0.

Model validation parameters are displayed in Figure 4A–D. For all four comparison groups, R^2^Y values exceeded 0.91 and Q^2^ values surpassed 0.75. Permutation testing further confirmed the absence of model overfitting, supporting satisfactory predictive reliability. Collectively, these results demonstrated that incremental exercise exerted a statistically significant effect on the plasma metabolome of Yili horses, with the magnitude of this effect progressively intensifying as exercise intensity increased.

### 3.5. Differential Metabolite Analysis

**Figure 5:**
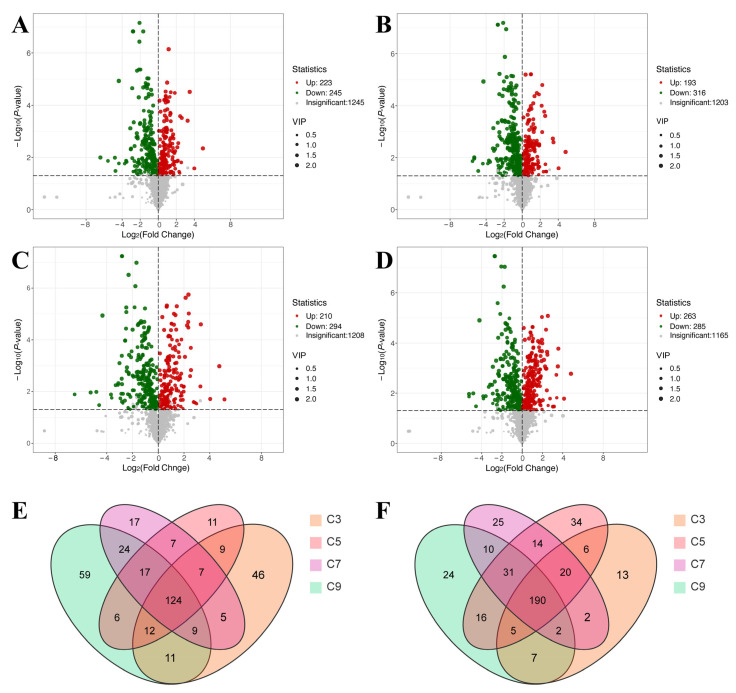
Comparative analysis of DEMs among groups. Note: (**A**) volcano plot of DEMs for C3 vs. C0; (**B**) volcano plot of DEMs for C5 vs. C0; (**C**) volcano plot of DEMs for C7 vs. C0; (**D**) volcano plot of DEMs for C9 vs. C0; (**E**) Venn diagram of the number of upregulated common DEMs among the four groups; (**F**) Venn diagram of the number of downregulated common DEMs among the four groups.

To characterize the impact of incremental exercise on the equine plasma metabolome, pairwise differential analyses were performed between each exercise group (C3, C5, C7, and C9) and the resting control (C0). These comparisons yielded 468, 509, 504, and 548 DEMs, respectively. Directional analysis revealed that, relative to C0, 223 metabolites were upregulated and 245 downregulated at C3; 193 upregulated and 316 downregulated at C5; 210 upregulated and 294 downregulated at C7; and 263 upregulated and 285 downregulated at C9 (Figure 5A–D and Tables S3–S6). Venn diagram analysis further identified a core set of 314 shared DEMs across all exercise intensities, consisting of 124 upregulated and 190 downregulated metabolites (Figure 5E,F).

To identify the principal metabolic drivers during progressive exercise, we prioritized the highest-intensity comparison (C9 vs. C0) and selected representative metabolites from three major categories—amino acids and their metabolites, organic acids and their derivatives, and fatty acyls—drawn from the shared pool of 314 DEMs. This targeted approach aimed to pinpoint the key metabolic nodes and core effector molecules underlying the intensity-dependent shifts in the equine plasma metabolome (Table 3). The absolute Cliff’s delta values for the fifteen differential metabolites ranged from 0.852 to 1.000, all exceeding the threshold for large effect sizes (|δ| ≥ 0.474). Furthermore, the 95% confidence intervals for all metabolites excluded zero, further corroborating the reliability of the observed between-group differences.

### 3.6. Pathway Enrichment Analysis

**Figure 6:**
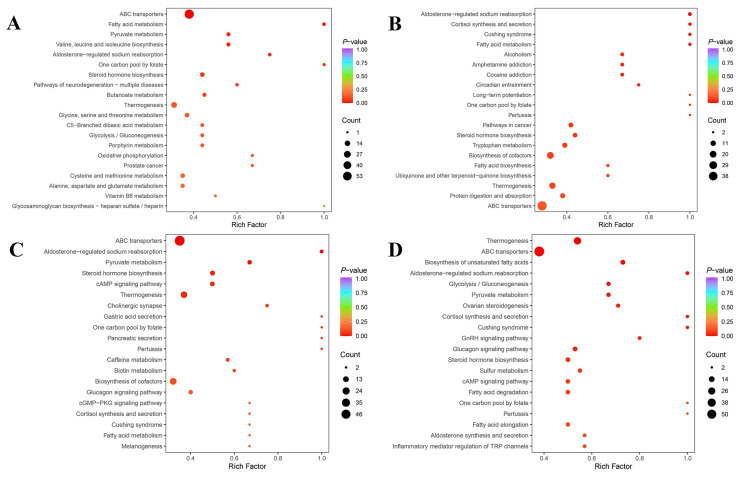
KEGG enrichment analysis of DEMs. Note: (**A**) KEGG enrichment analysis of C3 vs. C0; (**B**) KEGG enrichment analysis of C5 vs. C0 (**C**) KEGG enrichment analysis of C7 vs. C0; (**D**) KEGG enrichment analysis of C9 vs. C0.

Pathway enrichment analysis against the KEGG database identified five metabolic pathways that were commonly enriched across all four exercise comparisons (C3, C5, C7, and C9 vs. C0): ABC transporters, thermogenesis, aldosterone-regulated sodium reabsorption, steroid hormone biosynthesis, and one carbon pool by folate (Figure 6A–D).

### 3.7. Correlations Between Cardiac Structure and Differential Metabolites

Correlation analysis between cardiac structural parameters and amino acids and their metabolites revealed eighteen significant associations. At rest (0 m/s), LVFWd and LVFWs were highly significantly and positively correlated with proline-hydroxyproline (*p* < 0.01) and significantly and negatively correlated with N2-(1-carboxyethyl)-L-arginine (*p* < 0.05). At 9 m/s, LVFWs remained significantly positively correlated with proline-hydroxyproline (*p* < 0.05) and significantly negatively correlated with N2-(1-carboxyethyl)-L-arginine (*p* < 0.05), and they were also significantly positively correlated with Phe-Gly at rest (*p* < 0.05). LVIDs was significantly negatively correlated with N2-(1-carboxyethyl)-L-arginine at 9 m/s (*p* < 0.05) and significantly positively correlated with Gly-Phe at rest (*p* < 0.05).

Correlation analysis between cardiac structural parameters and organic acids and their derivatives identified six significant associations. IVSs was significantly negatively correlated with ferulic acid at 9 m/s (*p* < 0.05). LADd was significantly positively correlated with succinic acid at rest (0 m/s) (*p* < 0.05). At 9 m/s, MWTd, LVM, and LV MASS-I were each significantly positively correlated with succinic acid (*p* < 0.05), and LV MASS-I was also significantly positively correlated with methylmalonic acid (*p* < 0.05).

**Figure 7:**
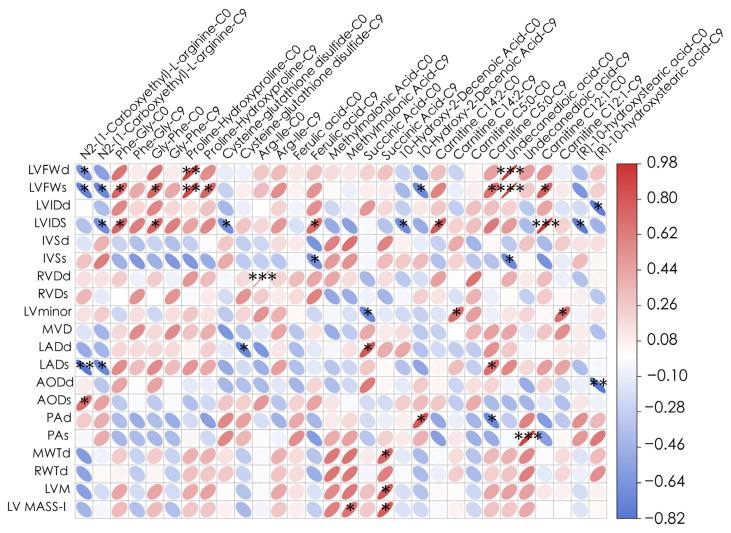
Correlation analysis between cardiac structure and DEMs. Note: The colors of the shapes within the grid indicate correlation: red for positive correlation, blue for negative correlation. The darker the color or the thinner the shape, the greater the absolute value of the correlation. Among them, “*” indicates *p* < 0.05, “**” indicates *p* < 0.01, and “***” indicates *p* < 0.001.

Correlation analysis between cardiac structural parameters and fatty acyls identified twelve significant associations. For instance, LVIDd was significantly negatively correlated with (R)-10-hydroxystearic acid at 9 m/s (*p* < 0.05). LVIDs showed a significant negative correlation with (R)-10-hydroxystearic acid at rest (0 m/s; *p* < 0.05), a highly significant positive correlation with carnitine C12:1 at 0 m/s (*p* < 0.001), and a significant positive correlation with carnitine C14:2 at 0 m/s (*p* < 0.05). Additionally, both LVFWd and LVFWs were highly significantly and positively correlated with undecanedioic acid at rest (0 m/s; *p* < 0.001) (Figure 7).

## 4. Discussion

### 4.1. Association Between Cardiac Structural Phenotype and Amino Acid Metabolic Dynamics During Incremental Exercise in Yili Horses

Proline-hydroxyproline emerged as a particularly informative marker. It displayed pronounced upregulation throughout the exercise protocol and maintained positive correlations with LVFWd and LVFWs at rest (0 m/s), as well as with LVFWs at peak workload (9 m/s). This finding resonates with established biochemical roles. Proline constitutes an obligate precursor for collagen and elastin synthesis [29]. Hydroxyproline serves as a definitive indicator of collagen turnover and extracellular matrix remodelling [30]. Prior investigations have substantiated the functional significance of this metabolic axis. Dietary supplementation with proline-enriched carbohydrate mixtures markedly prolongs exhaustion time and augments endurance performance in murine models [31]. Judicious exercise loading preserves collagen metabolic equilibrium in cardiac tissue [32]. Together, these reports affirm that collagen metabolism represents a fundamental determinant of myocardial wall structural integrity.

Against this backdrop, the observed upregulation of proline-hydroxyproline signifies an accelerated turnover of collagen matrix within the myocardium and its associated connective tissues during incremental exercise. This enhanced matrix maintenance provides the biochemical foundation for preserving tissue tensile strength. It enables the heart to resist excessive stretch or deformation during vigorous systolic contraction. In doing so, it furnishes critical metabolic support for cardiac structural stability and haemodynamic output under progressively increasing loads. Furthermore, the positive correlation between proline-hydroxyproline and left ventricular free wall thickness parameters suggests that horses possessing a thicker basal LVFW mount a more pronounced matrix maintenance response during acute exercise. This metabolic adaptation appears calibrated to match the escalating wall stress imposed by incremental workloads. It illuminates how intrinsic cardiac geometry directs acute amino acid metabolic strategies to safeguard myocardial mechanical competence under exercise stress.

N2-(1-Carboxyethyl)-L-arginine also warrants attention. It exhibited significant upregulation during incremental exercise and was negatively correlated with LVFWs and LADs at both 0 m/s and 9 m/s. This metabolite represents a glycated derivative of arginine. Its accumulation typically reflects enhanced glycolytic flux and the buildup of reactive carbonyl intermediates under high-intensity exercise [33]. Exercise-induced glycation stress is known to interact closely with the cardiac metabolic milieu [34]. The negative correlations observed in this study carry important physiological implications. Horses with thicker left ventricular free walls and larger left atrial diameters maintained lower circulating levels of this glycation product at rest and during high-speed exercise. This pattern suggests that superior cardiac geometry is accompanied by a more robust capacity to regulate and buffer glycolytic byproducts. Such metabolic control enables these individuals to preserve homeostasis more effectively when facing acutely incremental workloads.

Arg-Ile was significantly upregulated during incremental exercise and enriched in the ABC transporter pathway. Arginine participates in protein synthesis [35] and immune modulation [36]. Isoleucine, as a branched-chain amino acid, is preferentially utilised by skeletal muscle and contributes to fatty acid metabolism and glycaemic control [37,38]. The upregulation of Arg-Ile and its pathway enrichment indicate that transmembrane amino acid and peptide transport, substrate redistribution, and protein turnover were enhanced with increasing workload [16,39]. At rest, Arg-Ile exhibited a highly significant positive correlation with RVDd. This suggests that arginine-related haemodynamic regulation, together with the energetic support provided by branched-chain amino acids, facilitates right ventricular filling. Analogously, Phe-Gly and Gly-Phe were likewise enriched in the ABC transporter pathway and associated with structural indices including LVFWs and LVIDs. Collectively, these findings reveal that Yili horses with divergent cardiac structural phenotype mount distinct peptide transport and turnover responses under acutely incremental loads. The organism accelerates transmembrane allocation of amino acid substrates to secure the material and energetic resources necessary for myocardial structural stability and sustained contractile performance during high-intensity exercise.

GSSG is the oxidised form of glutathione (GSH), and the GSSG/GSH ratio serves as a critical index for assessing cellular redox status [40]. In the present study, GSSG levels rose significantly during incremental exercise and correlated negatively with LVIDs at 0 m/s and with LADd at 9 m/s. High-intensity exercise augments reactive oxygen species generation [41]. This drives the oxidation of GSH to GSSG and consequently depletes antioxidant reserves. The observed GSSG upregulation suggests that heightened oxidative stress may perturb the homeostatic stability of cardiac chamber dimensions. Moreover, previous work has demonstrated that post-exercise elevations in the GSSG/GSH ratio can precipitate muscular fatigue [42], underscoring the functional consequences of a compromised redox buffer system during strenuous exertion.

### 4.2. Association Between Cardiac Structural Phenotype and Organic Acid Metabolic Dynamics During Incremental Exercise in Yili Horses

Succinic acid is a pivotal intermediate of the tricarboxylic acid cycle. Its elevation generally reflects augmented mitochondrial oxidative flux [43]. Under high-intensity conditions, however, succinic acid accumulation may also indicate inadequate local oxygen supply and heightened oxidative stress [44]. In this study, succinic acid was markedly upregulated during incremental exercise and correlated positively with MWTd, LVM, and LV MASS-I at 9 m/s. These findings suggest that horses possessing greater left ventricular mass and thicker myocardial walls engage more vigorous oxidative metabolic mobilisation under acute high loads. This enables them to meet the energetic demands of sustained myocardial contraction. At the same time, the pronounced rise in succinic acid underscores the substantial escalation in cardiac energy requirements during incremental exercise. The accumulation of this metabolite represents an active oxidative metabolic recruitment, one calibrated to match progressively increasing workloads.

Methylmalonic acid is a catabolic product of odd-chain fatty acids and amino acids. Its elevation typically signals increased mitochondrial metabolic load and diminished energy utilization efficiency [45]. In this study, methylmalonic acid was significantly upregulated during incremental exercise and correlated positively with LV MASS-I. This result indicates that horses with greater left ventricular mass experience more pronounced energy metabolic turnover and mitochondrial stress during incremental exercise. The accumulation of this metabolite thus serves as a biochemical footprint of the augmented metabolic burden borne by hearts with larger structural mass under acutely increasing workloads.

Ferulic acid is a phenolic acid derivative with potent antioxidant, anti-inflammatory, and cardioprotective properties [46]. It exerts anti-fatigue effects through activation of antioxidant defense systems [47,48]. In the present study, ferulic acid declined significantly during incremental exercise. At 9 m/s, it correlated positively with LVIDs and negatively with IVSs. Similarly, 10-hydroxy-2-decenoic acid possesses anti-inflammatory and antioxidant activities [49]. It also decreased markedly during incremental exercise and correlated positively with PAd. The concurrent depletion of ferulic acid and 10-hydroxy-2-decenoic acid suggests that as exercise load escalates, rising reactive oxygen species generation and intensified inflammatory responses trigger preferential mobilization and consumption of these protective metabolites. This compensatory drain serves to preserve cardiovascular homeostasis and mitigate exercise-induced oxidative damage [50].

### 4.3. Association Between Cardiac Structural Phenotype and Fatty Acyl Metabolic Dynamics During Incremental Exercise in Yili Horses

Carnitine C14:2 and C12:1 are critical intermediates that shuttle long-chain fatty acids into mitochondria for β-oxidation [51,52]. Their upregulation reflects enhanced fatty acid mobilisation, transmembrane transport, and mitochondrial oxidative flux as workloads escalate [53]. In this study, both metabolites rose significantly during incremental exercise and correlated positively with LVFWs, LVIDs, LV minor, and RVDd. This pattern indicates that horses with thicker left ventricular walls and larger chamber dimensions possess a stronger capacity for fatty acid transport and β-oxidative energy supply [54]. Cardiac muscle relies heavily on fatty acid oxidation; thus, the elevation of these acylcarnitines signals not merely skeletal muscle substrate switching but also an augmented cardiac adaptive capacity under sustained exercise [55].

By contrast, carnitine C5:0 declined significantly during incremental exercise. At 9 m/s, it correlated positively with LVFWs and LADs, and negatively with PAd. Carnitine C5:0 is a short-chain incomplete oxidation product. Its circulating levels are inversely associated with mitochondrial protein synthesis, respiratory capacity, and maximal oxygen uptake [56]. The observed reduction therefore points to enhanced mitochondrial oxidative metabolism and improved substrate utilisation efficiency. In the present study, the decline in carnitine C5:0 suggests that Yili horses curtail short-chain incomplete oxidation products as exercise load increases. This metabolic shift toward more efficient fatty acid oxidation likely contributes to improved exercise performance.

Undecanedioic acid is a long-chain dicarboxylic acid belonging to the medium-to-long-chain dicarboxylic acid family. It is mainly derived from the ω-oxidation of long-chain fatty acids and the chain-shortening process in peroxisomes [57,58]. In this study, it rose significantly during incremental exercise and correlated positively with LVFWd and LVFWs at rest, and with PAs at 9 m/s. This suggests that horses with thicker myocardial walls recruited alternative lipid oxidative pathways to meet escalating energy demands during late-stage exercise.

(R)-10-hydroxystearic acid, a hydroxy fatty acid derivative, followed the opposite trajectory. It declined markedly during incremental exercise and correlated negatively with LVIDd and AODd. Hydroxy fatty acid derivatives participate in membrane lipid metabolism and redox homeostasis; their accelerated turnover helps buffer exercise-induced oxidative damage [59]. The pronounced decrease in horses with larger left ventricular and aortic dimensions indicates that a more favourable cardiac structural phenotype is accompanied by more vigorous lipid metabolic and antioxidant responses, preserving membrane stability and vascular function under high-intensity loads.

### 4.4. Integrated Metabolic–Structural Coupling During Incremental Exercise in Yili Horses

In summary, plasma metabolic dynamics and cardiac structural phenotype showed robust, multi-tiered coupling during incremental exercise. Amino acid metabolites supported myocardial stability through collagen turnover and redox regulation. Organic acids maintained cardiac homeostasis by mobilising mitochondrial energy and antioxidant defences. Fatty acyls fuelled sustained contraction via enhanced fatty acid oxidation. The shared enrichment of ABC transporter and thermogenesis pathways indicates that horses with different cardiac structural phenotypes activated coordinated multi-system metabolic responses. Specifically, proline-hydroxyproline, succinic acid, and carnitine C14:2 correlated positively with left ventricular wall thickness, suggesting that thicker-walled individuals exhibited distinctive signatures of active collagen turnover, augmented mitochondrial delivery, and intensified fatty acid oxidation. Greater left ventricular mass, meanwhile, accompanied more pronounced energy and lipid metabolic turnover. These concerted patterns reveal an intrinsic metabolome-geometry axis in exercising Yili horses [19].

Additionally, a complementary study was conducted concurrently with the present trial. This study measured plasma lactate concentrations in the same cohort of Yili horses during incremental treadmill exercise [60]. Lactate levels only increased significantly at the highest workload (9 m/s) compared with rest (0 m/s) and low-intensity exercise (3 m/s) (*p* < 0.01). No significant differences were detected among 0 m/s, 3 m/s, and 5 m/s. This dynamic trajectory of lactate across incremental workloads closely paralleled the metabolic transition patterns revealed by our broad-targeted metabolomics panel. Collectively, these findings provide independent corroboration of the staged metabolic response characteristic of incremental exercise.

The present study has several limitations. First, the sample size was small (*n* = 9), with horses recruited via convenience sampling, and no formal a priori sample size calculation (power analysis) was performed. Additionally, the absolute Cliff’s delta values for all differential metabolites in this study exceeded 0.85. Although this magnitude is consistent with the extreme physiological contrast between rest and near-maximal exercise, it may also partly reflect small-sample effect-size inflation. Under small-sample conditions, point estimates of effect size can be upwardly biased relative to the true values. However, even after applying a conservative downward correction of 20–30%, the effect sizes for all metabolites would still comfortably exceed the large-effect threshold (|δ| ≥ 0.474), indicating that the robustness of our core conclusions is not fundamentally compromised. Moreover, the 95% confidence intervals derived via bootstrap resampling under small-sample conditions should be interpreted as approximate indicators of estimation precision rather than as exact intervals. Consequently, the current findings should be regarded as exploratory, and their generalizability is therefore limited; validation in independent cohorts with larger sample sizes is warranted. Second, the CV for certain echocardiographic parameters exceeded 10%. Although triplicate measurements were obtained and averaged for each horse, inherent inter-individual variability, technical demands in acquiring standard imaging planes, and the potential leverage of outliers in a small sample may have introduced uncertainty into the parameter estimates. Third, exercise-induced hemoconcentration was not corrected using total plasma protein concentration, potentially biasing the interpretation of absolute plasma metabolite concentrations. Fourth, conventional exercise biomarkers (e.g., glucose, hematocrit) and cardiac-specific markers (e.g., CK-MB) were not assayed concurrently, nor were key amino acids released from skeletal muscle during exercise (e.g., alanine, glutamine) measured, which precluded direct quantitative comparison with prior studies.

Despite these limitations, this study provides preliminary quantitative insight into the metabolic–cardiac coupling in Yili horses during incremental exercise, with the following practical implications: (1) The dynamic changes in plasma metabolites were significantly associated with cardiac structural parameters, suggesting that trainers may utilize non-invasive echocardiography combined with blood metabolite monitoring as an integrative approach to assess exercise adaptation status under progressive workloads. (2) Given the pronounced inter-individual variability in certain echocardiographic indices (CV > 10%), veterinarians and trainers are advised to adopt standardized operating procedures in clinical assessment—including repeated measurements averaged by the same experienced operator—and to synthesize these data with established physiological markers (e.g., heart rate variability, lactate threshold) for comprehensive evaluation. Single echocardiographic measurements should not be used as absolute determinants in horse selection or as sole criteria for training efficacy. Future investigations should incorporate post-exercise recovery sampling, expand sample sizes through multi-center collaborations, and systematically integrate exercise biomarkers with cardiac-specific indicators to further validate and extend these preliminary observations.

## 5. Conclusions

This study integrates echocardiography with widely targeted metabolomics to decode the metabolic–structural coupling that governs cardiac performance in Yili horses during incremental exercise. The plasma metabolome exhibited pronounced intensity-dependent divergence from the resting state, yielding 314 core differential metabolites enriched in ABC transporter and energy metabolism pathways. Left ventricular wall thickness correlated with collagen turnover markers (proline-hydroxyproline), ventricular mass with tricarboxylic acid cycle intermediates (succinic acid), and chamber dimensions with long-chain fatty acid transporters (carnitine C14:2 and C12:1). These findings establish a coordinated metabolic–structural phenotype wherein amino acid, organic acid, and fatty acyl metabolites converge to sustain extracellular matrix renewal, mitochondrial energy delivery, and oxidative substrate utilisation. By associating cardiac geometry with specific circulating metabolic signatures, this study offers preliminary mechanistic insights into biomarker-based performance evaluation and precision training in equine sports science. Nevertheless, this conclusion remains to be further validated through conventional equine athlete assessments.

## Figures and Tables

**Table 1:** Cardiac structure indicators.

Indicator	Abbreviation
Left ventricular free wall thickness at end-diastole	LVFWd
Left ventricular free wall thickness at end-systole	LVFWs
Left ventricular end-diastolic dimension	LVIDd
Left ventricular end-systolic dimension	LVIDs
Interventricular septal thickness at end-diastole	IVSd
Interventricular septal thickness at end-systole	IVSs
Right ventricular end-diastolic dimension	RVDd
Right ventricular end-systolic dimension	RVDs
Left ventricular minor axis	LV Minor
Mitral valve diameter	MVD
Left atrial end-diastolic dimension	LADd
Left atrial end-systolic dimension	LADs
Aortic root end-diastolic dimension	AODd
Aortic root end-systolic dimension	AODs
Pulmonary artery end-diastolic dimension	PAd
Pulmonary artery end-systolic dimension	PAs
Mean left ventricular wall thickness at end-diastole	MWTd
Relative wall thickness at end-diastole	RWTd
Left ventricular mass	LVM
Left ventricular mass index	LV MASS-I

**Table 2:** Information on the structure of the horse’s heart.

Indicator (cm)	Horse ID									Mean	SD	CV
	111	120	78	115	8	20	47	79	49			
LVFWd	3.01	2.48	2.05	1.83	1.96	2.09	3.18	2.22	2.05	2.32	0.45	19.45
LVFWs	4.31	4.05	3.31	3.27	3.01	3.05	4.14	3.66	3.49	3.59	0.45	12.63
LVIDd	10.20	10.94	9.63	10.02	10.50	10.50	10.11	9.89	9.45	10.14	0.44	4.30
LVIDS	5.23	5.84	4.35	4.71	4.58	4.27	4.26	5.23	4.44	4.77	0.52	10.83
IVSd	2.05	2.26	2.75	3.79	2.35	3.27	3.44	3.31	2.70	2.88	0.57	19.67
IVSs	3.66	4.36	5.27	5.62	5.10	5.58	5.32	5.45	5.01	5.04	0.61	12.02
RVDd	2.62	2.66	2.66	2.57	2.61	1.92	2.66	2.31	2.22	2.47	0.25	10.05
RVDs	2.18	2.13	1.96	1.83	2.05	1.83	1.44	1.61	1.83	1.87	0.23	12.04
LVminor	16.22	14.82	15.39	16.12	15.61	14.58	14.99	15.47	15.34	15.39	0.52	3.37
MVD	10.16	9.65	8.98	9.67	9.80	9.68	9.57	9.33	9.77	9.62	0.31	3.19
LADd	10.40	10.49	9.57	10.27	10.19	11.09	11.34	10.45	11.19	10.55	0.53	5.02
LADs	9.68	8.95	8.02	8.87	8.42	9.45	9.31	9.79	9.00	9.05	0.55	6.02
AODd	4.65	5.22	4.42	4.65	5.21	5.28	4.81	4.91	4.61	4.86	0.29	6.04
AODs	6.14	6.94	7.15	6.58	6.94	6.05	6.24	6.12	6.72	6.54	0.39	6.02
PAd	4.40	3.43	4.94	3.89	4.28	5.15	3.99	4.14	3.71	4.21	0.52	12.42
PAs	5.62	4.82	5.68	5.45	5.19	5.67	5.50	5.31	5.18	5.38	0.27	4.99
MWTd	2.53	2.37	2.40	2.81	2.16	2.68	3.31	2.77	2.37	2.60	0.32	12.39
RWTd	0.50	0.43	0.50	0.56	0.41	0.51	0.66	0.56	0.50	0.51	0.07	13.30
LVM	2.58	2.64	2.18	2.92	2.16	2.94	3.79	2.80	2.09	2.68	0.50	18.73
LV MASS-I	0.46	0.50	0.44	0.58	0.45	0.59	0.74	0.57	0.41	0.53	0.10	18.52

**Table 3:** Differential metabolites screened in C9 vs. C0.

DEM	VIP	*p*-Value	FDR	Fold Change	Cliff’s Delta	95% CI	Type	Classification
N2-(1-Carboxyethyl)-L-Arginine	1.549	0.000	0.002	3.871	0.951	[0.778, 1.000]	up	Amino acids and their metabolites
Phe-Gly	1.913	0.000	0.002	2.035	1.000	[1.000, 1.000]	up	
Gly-Phe	1.913	0.000	0.002	2.035	1.000	[1.000, 1.000]	up	
Proline-Hydroxyproline	1.794	0.000	0.007	1.896	0.926	[0.704, 1.000]	up	
Cysteine-glutathione Disulfide	1.765	0.000	0.008	1.742	0.877	[0.556, 1.000]	up	
Arg-Ile	1.601	0.000	0.010	1.535	0.951	[0.778, 1.000]	up	
Ferulic Acid	2.166	0.000	0.000	0.152	−1.000	[−1.000, −1.000]	down	Organic acids and their derivatives
Methylmalonic Acid	2.220	0.000	0.007	5.495	1.000	[1.000, 1.000]	up	
Succinic Acid	2.178	0.001	0.015	5.209	1.000	[1.000, 1.000]	up	
10-Hydroxy-2-Decenoic Acid	1.522	0.002	0.023	0.205	−0.852	[−1.000, −0.519]	down	
Carnitine C14:2	1.888	0.000	0.002	5.795	1.000	[1.000, 1.000]	up	Fatty acyls
Undecanedioic Acid	1.752	0.000	0.002	5.122	0.975	[0.852, 1.000]	up	
Carnitine C5:0	2.106	0.000	0.002	0.421	−1.000	[−1.000, −1.000]	down	
Carnitine C12:1	1.976	0.000	0.004	3.865	0.975	[0.852, 1.000]	up	
(R)-10-Hydroxystearic Acid	1.515	0.000	0.004	0.3662	−0.901	[−1.000, −0.679]	down	

## Data Availability

The basic horse information and metabolomics data supporting the findings of this study are openly available in the figshare repository at https://doi.org/10.6084/m9.figshare.32085222.

## Supplementary Materials

The following supporting information can be downloaded at: https://www.mdpi.com/article/10.3390/ani16111672/s1, Table S1: Basic information of horses; Table S2: Details of the reagents used in the experiment; Table S3: C3 vs. C0 DEMs; Table S4: C5 vs. C0 DEMs; Table S5: C7 vs. C0 DEMs; Table S6: C9 vs. C0 DEMs.

## Abbreviations

The following abbreviations are used in this manuscript:

DEMs	Differential Expressed Metabolites
GSSG	Cysteine-Glutathione Disulfide
2D	Two-Dimensional
SD	Standard Deviation
CV	Coefficients of Variation
QC	Quality Control
KEGG	Kyoto Encyclopedia of Genes and Genomes
PCA	Principal Component Analysis
OPLS-DA	Orthogonal Partial Least Squares Discriminant Analysis
VIP	Variable Importance in Projection
FC	Fold Change
GSH	Glutathione
LVFWd	Left Ventricular Free Wall Thickness at End-Diastole
LVFWs	Left Ventricular Free Wall Thickness at End-Systole
LVIDd	Left Ventricular End-Diastolic Dimension
LVIDs	Left Ventricular End-Systolic Dimension
IVSd	Interventricular Septal Thickness at End-Diastole
IVSs	Interventricular Septal Thickness at End-Systole
RVDd	Right Ventricular End-Diastolic Dimension
RVDs	Right Ventricular End-Systolic Dimension
LV Minor	Left Ventricular Minor Axis
MVD	Mitral Valve Diameter
LADd	Left Atrial End-Diastolic Dimension
LADs	Left Atrial End-Systolic Dimension
AODd	Aortic Root End-Diastolic Dimension
AODs	Aortic Root End-Systolic Dimension
PAd	Pulmonary Artery End-Diastolic Dimension
PAs	Pulmonary Artery End-Systolic Dimension
MWTd	Mean Left Ventricular Wall Thickness at End-Diastole
RWTd	Relative Wall Thickness at End-Diastole
LVM	Left Ventricular Mass
LV MASS-I	Left Ventricular Mass Index
